# A Novel Characteristic Frequency Bands Extraction Method for Automatic Bearing Fault Diagnosis Based on Hilbert Huang Transform

**DOI:** 10.3390/s151127869

**Published:** 2015-11-03

**Authors:** Xiao Yu, Enjie Ding, Chunxu Chen, Xiaoming Liu, Li Li

**Affiliations:** 1 IOT Perception Mine Research Center, China University of Mining and Technology, Xuzhou 221000, China; E-Mails: db14060013@cumt.edu.cn (X.Y.); tb14060015@cumt.edu.cn (C.C.); lxm0779@cumt.edu.cn (X.L.); 2 School of Information and Electrical Engineering, China University of Mining and Technology, Xuzhou 221000, China; 3 School of Medicine Information, Xuzhou Medical College, Xuzhou 221000, China; 4 School of Mechanical and Electrical Engineering, Central South University, Changsha 410000, China; E-Mail: lilicsuee@163.com

**Keywords:** fault diagnosis, Hilbert–Huang Transform, salient features extraction, support vector machine, characteristic frequency bands

## Abstract

Because roller element bearings (REBs) failures cause unexpected machinery breakdowns, their fault diagnosis has attracted considerable research attention. Established fault feature extraction methods focus on statistical characteristics of the vibration signal, which is an approach that loses sight of the continuous waveform features. Considering this weakness, this article proposes a novel feature extraction method for frequency bands, named Window Marginal Spectrum Clustering (WMSC) to select salient features from the marginal spectrum of vibration signals by Hilbert–Huang Transform (HHT). In WMSC, a sliding window is used to divide an entire HHT marginal spectrum (HMS) into window spectrums, following which Rand Index (RI) criterion of clustering method is used to evaluate each window. The windows returning higher RI values are selected to construct characteristic frequency bands (CFBs). Next, a hybrid REBs fault diagnosis is constructed, termed by its elements, HHT-WMSC-SVM (support vector machines). The effectiveness of HHT-WMSC-SVM is validated by running series of experiments on REBs defect datasets from the Bearing Data Center of Case Western Reserve University (CWRU). The said test results evidence three major advantages of the novel method. First, the fault classification accuracy of the HHT-WMSC-SVM model is higher than that of HHT-SVM and ST-SVM, which is a method that combines statistical characteristics with SVM. Second, with Gauss white noise added to the original REBs defect dataset, the HHT-WMSC-SVM model maintains high classification accuracy, while the classification accuracy of ST-SVM and HHT-SVM models are significantly reduced. Third, fault classification accuracy by HHT-WMSC-SVM can exceed 95% under a *Pmin* range of 500–800 and a *m* range of 50–300 for REBs defect dataset, adding Gauss white noise at Signal Noise Ratio (SNR) = 5. Experimental results indicate that the proposed WMSC method yields a high REBs fault classification accuracy and a good performance in Gauss white noise reduction.

## 1. Introduction

Because roller element bearings (REBs) are a key part of mechanical equipment, their fault diagnosis is essential for safe operation of equipment [1]. In general, REBs fault diagnosis consists of three steps: vibration signal acquisition, fault features extraction from vibration signal, and fault type identification based on extracted fault features [2]. Vibration signals that represent fault features are widely used to monitor the condition of mechanical equipment. However, the vibration signal exhibits strong non-linear and non-stationary characteristics, due to the complexity of structure and activity. Therefore, extraction approaches for fault features from vibration signals that accurately reflect the bearing’s status are key for REBs fault diagnosis [3].

Since information about REBs condition in vibration signals is often hidden by impertinent vibration-related components, such as load, friction, clearance, stiffness and random noise, it is difficult to identify the REBs work state only by time or frequency domain features of vibration signals [4]. Hence, time–frequency analysis methods have been applied to fault features extraction of non-linear and non-stationary vibration signals, such as Wavelet Transform (WT) [3,5,6,7], Short Time Fourier Transform (ST) [8], Hilbert–Huang Transform (HHT) [9,10,11,12] and so on. WT is a good choice to provide time–frequency domain features of vibration signals. However, WT is a parametric method, meaning appropriate wavelet basis function and decomposition layer need to be selected for different applications [13,14]. Moreover, WT produces inevitable energy leakage when used for time–frequency signal analysis. Consisting primarily of empirical mode decomposition (EMD) and Hilbert spectral analysis, the HHT method is a non-parametric, time–frequency analysis method [15,16]. EMD is a self-adaptive approach, making it highly suitable and attractive for non-linear and non-stationary vibrations signals analysis, and thus has been widely applied in REBs fault diagnosis [17,18,19]. However, some challenges exist in the application of EMD to signal processing, such as over envelope, owing envelope, end effects and mode mixing [19,20,21].

Various artificial intelligence techniques have been used in machinery fault diagnosis [6,8,12,22,23,24,25], such as hidden Markov models (HMM), artificial neural networks (ANN) and support vector machines (SVM). ANN has experienced the fastest development over the past few years. Nevertheless, there are some drawbacks to neural networks, such as structure identification difficulties, Orthogonal Weight Estimators learning, local convergence, and poor generalization abilities [26,27]. Owing to its superior generalization capability, SVM shows good performance in solving problems like small sample size, as well as being capable of both non-linear and high-dimensional pattern recognition [28,29]. SVM solves over-fitting acceptably, as well as the local optimal solution problems of ANN. Recently, SVM has been popular in fault diagnosis of rotating machinery [30,31,32,33].

Until now, a variety of time domain, frequency domain and time–frequency domain statistical characteristics of vibration signals have been calculated to represent fault types, such as Root Mean Square, Standard Deviation, Kurtosis, Skewness, Shape Factor, Energy Entropy and so on. In [24], ten time–domain statistical characteristics and the energy entropies of Intrinsic Mode Functions (IMFs) were chosen as fault features to train an ANN for the bearing defects diagnosis. In [17], 21 time–domain statistical characteristics were extracted from different IMFs as the feature vectors. Then, principle component analysis (PCA) process was employed to extract the dominant components from said characteristics for gear faults detection. In [18], sixteen time–domain statistical characteristics and thirteen frequency–domain statistical characteristics were calculated from vibration signal IMFs, on which distance evaluation technique was used to select the salient features for improvement of classification accuracy for gear case abnormalities. In [30], two time–domain and two frequency–spectrum statistical characteristics are selected as the features to train the SVM with a novel hybrid parameter optimization algorithm for fault diagnosis of the rolling element bearings. In [6], the statistical parameters of the wavelet coefficients in 1–64 scales were calculated for the vibration signal. Then, statistical features in optimal scales (17–40) were extracted as inputs for ANN fault diagnosis classifier based on the Energy to Shannon Entropy Ratio. The feature extraction methods in above-described studies are all based on statistical characteristics. However, statistical characteristics can only represent certain characteristics of fault signals, which would lead to loss of the detailed global and local waveform characteristics. By use of statistical characteristic methods, fault classification accuracy may decrease with increased fault types.

In this article, entire HMS is attempted as REBs fault-classifier input vector, for which experiment results support its feasibility for REBs fault diagnosis. Nonetheless, the entire HMS contains too many components, possibly reducing classification efficiency of the fault diagnosis model. On the one hand, entire HMS contains noise and redundant components, which may lead to decreased classification accuracy; on the other hand, too many inputs will increase computational cost of the classifier. For entire HMS, therefore, a supervised feature extraction method named Window Marginal Spectrum Clustering (WMSC) is proposed for selection of characteristic frequency bands (CFBs), sliding window is used to divide the entire HMS into spectral bands, where upon the Rand Index (RI) criteria of clustering method is adopted to evaluate band. These bands with higher RI are selected to construct CFBs. The marginal spectrum components under CFBs (HMS-CFBs) are more fault patterns sensitive since the redundant and noise components can be filtered. A new, intelligent REBs fault diagnosis scheme (HHT-WMSC-SVM) is built next, based on HHT, WMSC and SVM, with HMS-CFB as the classifier input. Finally, experiments are carried out to verify the effectiveness of HHT-WMSC-SVM, in which inner race faults (IRF), outer race faults (ORF) and ball faults (BF) of bearings are considered to differing degrees.

Section 2 details the fundamental theory of HHT and SVM, while Section 3 presents the proposed novel WMSC method, including description of CFBs extraction procedure and REBs fault diagnosis. Following, experimental results and discussions are presented, including description of the experimental test bench, comparison of methods with statistical characteristics and effects of different parameters used in the model.

## 2. Theoretical Background

### 2.1. Empirical Mode Decomposition (EMD)

Based on local characteristics of signals in different time scales, EMD decomposes the signals into a set of complete and nearly orthogonal IMFs, each IMF corresponding to the vibration mode of a specific signal at a discrete frequency [15]. To deal with a non-stationary signal smoothly, an IMF is a function that satisfies two conditions. (1) In the whole data set, the number of extrema and the number of zero crossings must either equal or differ at most by one; (2) At any point, the mean values of the envelope defined by the local maxima and the envelope defined by the local minima are both zero.

The specific description of EMD for *x*(*t*) is presented as follows [15]:

(1) Obtain the local maxima and minima of *x*(*t*).
(2) Produce the upper and lower envelopes in accordance with the local maxima and the local minima of *x*(*t*).
(3) Their mean is designated as *m*_1_(*t*), and the difference between *x*(*t*) and *m*_1_(*t*) is the first component *h*_1_(*t*), *h*_1_(*t*) = *x*(*t*) − *m*_1_(*t*).
(4) Generally, *h*_1_(*t*) satisfies the conditions requisites for IMF, thus *h*_1_(*t*) can be treated as the first IMF component of *x*(*t*). If *h*_1_(*t*) does not satisfy the conditions for IMF, *h*_1_(*t*) can be treated as a new original signal. By repeating the above Steps (1)–(3), we obtain *h*_1_(*t*) and *m*_11_(*t*). *h*_11_(*t*) = *h*_1_(*t*) − *m*_11_(*t*). Repeat this sifting procedure *k* times, till *h*_1k_(*t*) is an IMF, which meets the criterion, *c*_1_(*t*) = *h*_1k_(*t*). Next, isolate *c*_1_(*t*) from *x*(*t*) by *r*_1_(*t*) = *x*(*t*) − *c*_1_(*t*), where *r*_1_(*t*) is the residue, treating this as new data, which meets *x*(*t*) = *r*_1_(*t*).
(5) Repeat the above Steps (1)–(4), until the original signal is decomposed into n IMFs, or when the residue *r_n_*(*t*) becomes smaller than the predetermined value, or the residue *r_n_*(*t*) becomes a monotonic function. Thus the EMD process is completed. After decomposition, *x*(*t*) can be expressed as
  (1) $$x(t)=\sum_{i=1}^{n}c_{i}(t)+r(t)$$

### 2.2. The Hilbert Spectrum

Selected in the previous section, these IMFs, *c_i_*(*t*), reflect the characteristics of the original signal in different time scales. At this point, perform Hilbert transform on each *c_i_*(*t*) as per:

(2) $$H\left(c_{i}(t)\right)=\frac{1}{\pi }\int_{-\infty }^{\infty }\frac{c_{i}(\tau )}{t-\tau }d\tau$$

The analytic signal of the original signal *z_i_*(*t*) can be expressed as:

(3) $$z_{i}\left(t\right)=c_{i}\left(t\right)+jH[c_{i}\left(t\right)]=a_{i}\left(t\right)e^{j\varphi_{i}\left(t\right)}$$

wherein the amplitude function of *a_i_*(*t*) is obtained by the equation,

(4) $$a_{i}\left(t\right)={\left[{c_{i}}^{2}\left(t\right)+H^{2}[c_{i}\left(t\right)]\right]}^{1/2}$$

Next, the phase function of *φ_i_*(*t*) can be defined as

(5) $$\varphi_{i}\left(t\right)=\arctan\left(\frac{H[c_{i}(t)]}{c_{i}(t)}\right)$$

from which the instantaneous frequency can be defined through further calculation;

(6) $$\omega_{i}(t)=\frac{d\varphi_{i}\left(t\right)}{dt}$$

Based on these equations, the original signal can be expressed as thus:

(7) x(t)=Re∑i=1nai(t)exp(jφi(t)dt)

Here, the residual term *r*(*t*) is ignored and Re reflects the actual element addressed, for which the Hilbert spectrum can be determined by the following:

(8) H(ω,t)=Re∑i=1nai(t)exp(j∫ωi(t)dt)

While the HMS can be defined by an integrated spectrum with respect to time as:

(9) $$h(w)=\int_{0}^{T}H(w,t)dt$$

The value of the HMS is a measure of total amplitude from each frequency, *w*, in different time scales. It represents amplitude changing with frequency across the entire frequency range and reflects whether the signal actually contains a given frequency [15,16]. When the machinery is under favorable conditions, the energy of the vibration signal spectrum mainly aggregates in the low-frequency region. While it aggregates in the high-frequency region, the machinery is likely under poor conditions.

For the reason of over envelope, owing envelope, mode mixing and noise, there are also some pseudo IMFs that may further interfere with fault diagnosis, necessitating an IMF selection method that removes these components. Correlation analysis has clear physical meaning, so correlation coefficients are employed to select fault-related IMFs, which coefficient between the original data *x*(*t*) and IMF component *c_i_*(*t*) can be presented as follows

(10) $$\rho_{\left(c_{i}(t),x(t)\right)}=\frac{E\left(c_{i}(t)\cdot x(t)\right)-E\left(c_{i}(t)\right)E\left(x(t)\right)}{\sqrt{D\left(c_{i}(t)\right)}\cdot \sqrt{D\left(x(t)\right)}}$$

where *E*(·) is the signal expectation and *D*(·) is the signal variance. The IMF components with greater correlation coefficient values are selected to calculate the HMS by Equations (7)–(9) in this article.

### 2.3. Support Vector Machine (SVM)

Support vector machine (SVM) is a statistical classification method based on the structural risk minimization approach, proposed by Vapnik *et al.* [28]. The basic principle of SVM is that it can find the optimal separating hyperplane that minimizes the upper bound of the generalization error by maximizing the margin between the separating hyperplane and the nearest sample points.

Said process may be described as a set of given *N* training data points $\left\{\left(x_{i},y_{i}\right)|x_{i}\in R^{n},y_{i}\in \left\{-1,+1\right\}\right\},i=1,\cdots ,N$, where *x_i_* is the input vector, *y_i_* is the label and *N* is the number of data samples. The sample space can be mapped on a high-dimensional feature space by non-linear mapping function *φ*(*x*) and the maximum margin separating hyperplane can be presented as *wφ*(*x*) + *b*, where *w* is the normal direction of a separation plane, and b is the scalar. The distance between the closest sample points and a separation plane is 1/ǁ*w*ǁ; thus, maximizing 1/ǁ*w*ǁ is equivalent to minimizing ǁ*w*ǁ. The problem of constructing an optimal hyperplane can be transformed into the following quadratic optimization solution:

(11) $$\underset{w,b}{\min}\frac{1}{2}{\left\|w\right\|}^{2}+C\sum_{i=1}^{n}\xi_{i}$$

with

$$Restrictions：y_{i}\left(w\phi (x_{i})+b\right)\geq 1-\xi_{i}\;and\;\xi_{i}\;\geq 0\;for\;i=1,\cdots ,n$$

where $\xi_{i}$ represents positive slack variables that are necessary to allow misclassification, and *C* imposes a trade-off between training error and generalization. By using the duality theory of optimization, the final decision function can be presented as:

(12) $$\begin{array}{l} f\left(x\right)=\mathrm{sgn}\left(\sum_{x_{i}\in sv_{s}}y_{i}a_{i}\langle \phi \left(x_{i}\right),\phi \left(x\right)\rangle +b\right)\\ \;=\mathrm{sgn}\left(\sum_{x_{i}\in sv_{s}}y_{i}a_{i}K\left(x_{i},x\right)+b\right) \end{array}$$

where *α_i_* symbolizes Lagrange multipliers, which can be determined during optimization process. $K(x_{i},x)=\langle \varphi \left(x_{i}\right),\varphi \left(x\right)\rangle$ is a kernel function, which allows access to spaces of high dimensions without the need to know the mapping function explicitly. A typical kernel function [30] offers these choices:

(1) Linear kernel, $K(x_{i},x)=<x_{i},x>$,
(2) Polynomial kernel, $K(x_{i},x)={\left(\gamma <x_{i},x>+\tau \right)}^{g}$,
(3) Radial Basis Function (RBF) kernel,
  (13) $$K(x_{i},x)=\exp(-\frac{{\left\|x_{i}-x\right\|}^{2}}{g^{2}}),g>0$$
(4) Sigmoid kernel, $K(x_{i},x)=\tanh(<x_{i},x>+\tau )$,

where *γ*, *τ* and *g* are kernel parameters for these kernel functions.

Since the RBF kernel can represent the complex non-linear relationships between the input vector and output value effectively by mapping the sample set into a high-dimensional feature space, we select it here as kernel function. The tradeoff Variable, *C*, and the kernel width, *g*, should be properly set for SVM by using the RBF kernel. As mentioned above, SVM was originally designed for binary classification. However, REBs fault detection is a multi-class pattern recognition task, which can be generally solved by decomposing the multi-class problem into several binary class problems [33]. The multi-class patterns recognition was handled by the “one-against-one” approach [34], in which a SVM is design between any two classes of samples in this article. Therefore, the *k* class samples need to design *k*(*k* − 1)/2 SVMs. When classifying an unknown sample, more than one classification functions need to be calculated. And finally the category that gets the most votes is the category of unknown sample.

## 3. Proposed Method for REBs Fault Detection

### 3.1. Problem Description

In current research, statistical characteristics of vibration signals within time–domain, frequency–domain or time–frequency–domain are applied to describe REBs fault types, such as range, mean value, standard deviation, skewness, kurtosis, crest factor, *etc.* [6,30]. Statistical characteristics that can show partial features of the vibration signals are used as the input of fault classifiers for classifier model training and fault classification. However, we cannot achieve overall description of the signal from the statistical characteristics, especially for the local waveform features that contain vital diagnosis information. As the input for fault classifiers that recognize REBs fault types, the HMS of the vibration signal is here applied in lieu of statistical characteristics. Detailed in Section 4.2, results demonstrate that fault classification via HMS input vector does effectively and favorably detect REBs fault types when compared to the statistical characteristics method.

HMS describes the time–frequency characteristics of the vibration signal, immensely impacting fault-type detection, while HMS does contain copious redundant information, thus expanding space to be investigated and increasing the calculation complexity for the classifier. In addition, within regions of frequency bands with indistinct features and noise, accuracy of classification is reduced. To improve the effectiveness of REBs fault diagnosis, proposed here is a sensitive characteristic–frequency band selection method, named as WMSC, based on a sliding window and RI criteria of clustering method.

### 3.2. Feature Extraction Method WMSC

In WMSC, the HMS of each training sample is divided into multiple sub-HMS windows. Then the sub-HMS windows set under the same frequency band are clustered by K-means method, from which the RI of the clustering results becomes the evaluation index of each sub-HMS windows set. The CFBs can be obtained by stacking the frequency bands of the windows sets with possessing a greater RI value. The specifics for evaluating WMSC are summarized the following steps.

**Step 1.** In the training dataset, there are *M* kinds of REBs fault types in the training dataset, and *N* vibration signal samples in each type of REBs fault pattern. The HMSs set of fault samples, ***MSP***, can be expressed as

$$MSP=\left[\begin{array}{llll} SP_{11} & SP_{12} & \cdots & SP_{1N}\\ SP_{21} & SP_{22} & \cdots & SP_{2N}\\ \;\vdots & \;\vdots & \ddots & \vdots \;\\ SP_{M1} & SP_{M2} & \cdots & SP_{MN} \end{array}\right]$$

where ***SP_ij_*** is the *j*th sample HMS of *i*th kind of REBs fault type, which can be defined in turn as *SP* = [*sp_1_*, *sp_2_*,…, *sp_i_*], where *l* is the frequency component number of ***SP_ij_***. Starting from the first component, ***SP_ij_*** can be divided into an *l − m + 1* sub-HMS windows set, ***W****_ij_* = [***W****_ij_^1^*, ***W****_ij_^2^*, …, **W***_ij_^l-m+1^*], by a sliding window with a window size of *m* frequency components and step size of one frequency component. The relationship between ***W_ij_^k^*** and ***SP_ij_*** is described by

$${W_{ij}}^{k}=[sp_{k},sp_{k+1},\ldots ,sp_{k+m-1}]$$

After implement above procedure to ***MSP***, windows sets ***MW*** are obtained as:

$$MW=\left[\begin{array}{llll} W_{11} & W_{21} & \cdots & W_{1N}\\ W_{21} & W_{21} & \cdots & W_{2N}\\ \;\vdots & \;\vdots & \ddots & \vdots \;\\ W_{M1} & W_{M2} & \cdots & W_{MN} \end{array}\right]$$

**Step 2.** By extracting the sub-HMS windows from the same frequency band, we can obtain *l + m* *− 1* frequency band sub-HMSs windows sets, [***MW****^1^*, ***MW****^2^*,…, ***MW****^l − m + 1^*], where ***MW^k^*** can be expressed by:

$$MW^{k}=\left[\begin{array}{llll} W_{11}^{k} & W_{12}^{k} & \cdots & W_{1N}^{k}\\ W_{21}^{k} & W_{22}^{k} & \cdots & W_{2N}^{k}\\ \;\vdots & \;\vdots & \ddots & \vdots \;\\ W_{M1}^{k} & W_{M2}^{k} & \cdots & W_{MN}^{k} \end{array}\right]$$

Next, classify ***MW^k^*** into *h* clustering partitions using the k-means method, where *h* is the fault labels. To judge the accuracy of clustering results, we will calculate the RI [35,36] of the clustering partitions.

Given a set of n objects **X** = {*x*_1_, *x*_2_,_…_, *x*_n_}, suppose **P** = {*p*_1_, *p*_2_,⋯, *p*_n_} and **Q** = {*q*_1_, *q*_2_,…, *q*_n_} represent classes of the objects by k-means algorithm and real class memberships, respectively. The RI, *rand*, is then defined as:

(14) rand=(a+b)/(a+b+c+d)

where:

a: number of object pairs {*x_i_, x_j_*} belonging to the same class in ***Q*** and to the same class in ***P***.
b: number of object pairs {*x_i_, x_j_*} belonging to the same class in ***Q*** and to different classes in ***P***.
c: number of object pairs {*x_i_, x_j_*} belonging to different classes in ***Q*** and to the same class in ***P***.
d: number of object pairs {*x_i_, x_j_*} belonging to different classes in ***Q*** and to different classes in ***P***.

RI measures the degree of similarity between the obtained partition and the true clustering structure underlying the data between 0 and 1, where 0 indicates complete disagreement and 1 indicates complete agreement. Necessarily, the greater the value of *Rand*, the better the clustering performance will be.

Once clustering analysis is performed for the *l − m + 1* frequency band sub-HMSs windows sets [***MW****^1^*, ***MW****^2^*, …, ***MW****^l−m+1^*], we can obtain the RI sequence ***Rand_MW*** *=* {*rand(1)*, *rand(2)*, …, *rand(l-m+1)*}. In this article, we presume that the greater the value of *rand(k)* is, the better the spectrum information in ***MW^k^*** will reflect true fault characteristics. Therefore, the frequency bands of the windows sets with greater RI should be selected to construct the CFBs *a priori*.

**Step 3.** The frequency band of ***MW^k^*** is stacked in frequency components set, ***S***, one by one according to the order of ***Rand_MW*** from great to small, while the overlapping frequency components should be recorded only one time. The process of superposition will stop when the number of frequency components in ***S*** is greater than threshold parameter *Pmin*, upon where the CFBs, ***S***, can be obtained. Those HMS components under CFBs (HMS-CFBs) will be used as the new input to the fault classifier.

### 3.3. Proposed REBs Fault Detection Model

**Figure 1:**
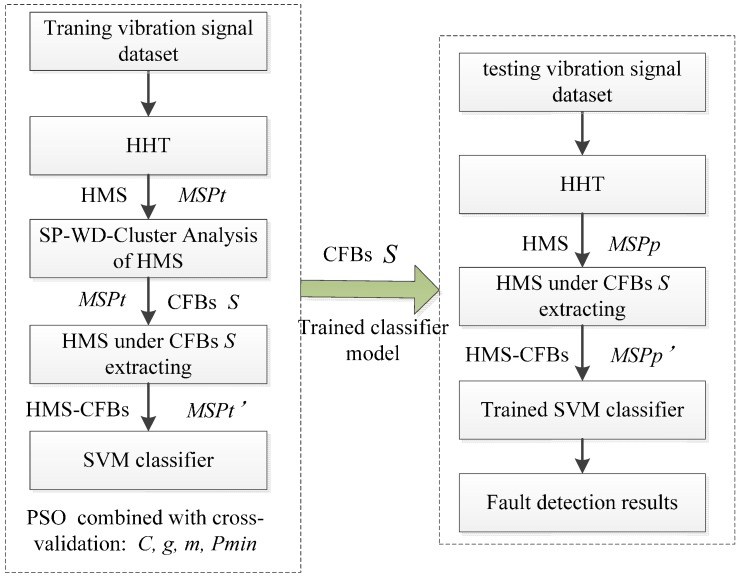
The procedure for the HHT-WMSC-SVM REBs fault detection model.

Based on HHT, SVM and WMSC, the multi REBs fault patterns detection model, named as HHT-WMSC-SVM, is shown as Figure 1. Initially, the HMSs set ***MSPt*** can be obtained by implementing HHT in the training dataset, from which the CFBs ***S*** of ***MSPt*** is obtained using WMSC. The HMS-CFBs set ***MSPt’*** is extracted from ***MSPt*** by CFBs ***S***. After training the SVM classifier model using ***MSPt’*** and fault type labels, the trained SVM-classifier model is constructed. At last, the HMSs set of testing dataset ***MSPp*** can be obtained by applying HHT. The HMS-CFBs ***MSPp’*** is extracted from ***MSPp*** by CFBs ***S***, which is obtained from training dataset. The fault type of testing dataset sample can be detected via the trained SVM-classifier model with ***MSPp’*** as inputs. As established, the penalty factor *C* and kernel parameter *g* will affect the performance of SVM-classifier. Meanwhile, window size *m* and the minimum frequency components threshold *Pmin* in the WMSC method will also affect the fault detection effectiveness of the HHT-WMSC-SVM model. Therefore, *m*, *Pmin*, *C* and *g* are set as four parameters for the HHT-WMSC-SVM model, while the PSO method combined with cross-validation is applied for obtaining the optimal parameters.

## 4. Experimental Result and Analysis

Rolling element bearing is one of the most important and common components in rotary machines and bearings failures may lead to fatal breakdowns of machines and can force unacceptably long time maintenance stops. Fast, accurate and ready detection of the existence and severity of bearing faults is therefore critical. In order to implement and evaluate the proposed WMSC-based REBs fault diagnosis model, we conducted three groups of experiments. In the first group, three REBs fault defects datasets served as accuracy test cases in HHT-WMSC-SVM model fault classification. To test the anti-noise ability of the HHT-WMSC-SVM model in second experiment set, Gauss white noise at different SNRs were added to the REBs fault defect dataset. In the third group, the effects of parameters *m* and *Pmin* in WMSC method on the fault classification performance were analyzed by REBs defect dataset adding Gaussian white noise at SNR = 5. For comparison, HHT-SVM and ST-SVM models were additionally employed in the first and second groups of experiments.

### 4.1. Experiment Setup

The bearing fault signals used in this article come from the bearing data center of Case Western Reserve University (CWRU) [37]. The bearings used in this work are deep groove ball bearings of the type 6205-2RS JEM SKF at DE and 6203-2RS JEM SKF at FE. Single point faults with fault diameters from 0.007 in to 0.040 in in diameter were introduced separately at the inner raceway, rolling element and outer raceway. Vibration data were recorded for motor loads of zero to three horsepower (motor speeds of 1797 to 1720 RPM).

The accelerometer data at DE are used as original signals for the detection of four kinds of DE motor housing REB conditions, namely: healthy bearing (HB), IRF, ORF and BF. In each fault pattern, 60 samples are acquired from vibration signal in time domain, while each sample contains 2000 continuous data points.

The first two datasets listed in Table 1, A and B, are used for analysis here, where 20 samples of each fault pattern are randomly designated as the training samples, and the remaining 40 samples serve as testing samples. Contained in dataset A are different bearing defect locations at the same level of defect severity (0.007 in), while dataset B comprises different bearing defect locations at differing levels of defect severity (0.007in and 0.014 in). As shown in Table 1, there are 240 samples in dataset A (80 samples in the training set, and the remaining 160 samples in the testing set) and 420 samples in dataset B (140 samples in the training set, and the remaining 280 samples in testing set).

**Table 1:** Detailed specifics of REB fault datasets A and B.

Dataset	Condition	Defect Size (in)	Rotating Speed	Training Samples	Testing Samples	Label
A	HB	-	1730	20	40	1
	IRF	0.007	1730	20	40	2
	BF	0.007	1730	20	40	3
	ORF	0.007	1730	20	40	4
B	HB	-	1730	20	40	1
	IRF	0.007	1730	20	40	2
	IRF	0.014	1730	20	40	3
	BF	0.007	1730	20	40	4
	BF	0.014	1730	20	40	5
	ORF	0.007	1730	20	40	6
	ORF	0.014	1730	20	40	7

It is useful for many applications to recognize the “incipient” REBs fault patterns with the model achieved by the “severe” REBs fault data. In order to test the model adaptability in this case, the third dataset, C, are used for analysis here. As show in Table 2, samples in different bearing defect locations at defect size of 0.014 in are employed as the training set, while the 0.007 in are employed as the testing set.

**Table 2:** Detailed specifics of REB fault dataset C.

Dataset	Condition	Rotating Speed	Training Samples/Defect Size (in)	Testing Samples/Defect Size (in)	Label
C	HB	1730	20/-	40/-	1
	IRF	1730	20/0.014	40/0.007	2
	BF	1730	20/0.014	40/0.007	3
	ORF	1730	20/0.014	40/0.007	4

### 4.2. Experimental Validation of the Proposed Method

**Figure 2:**
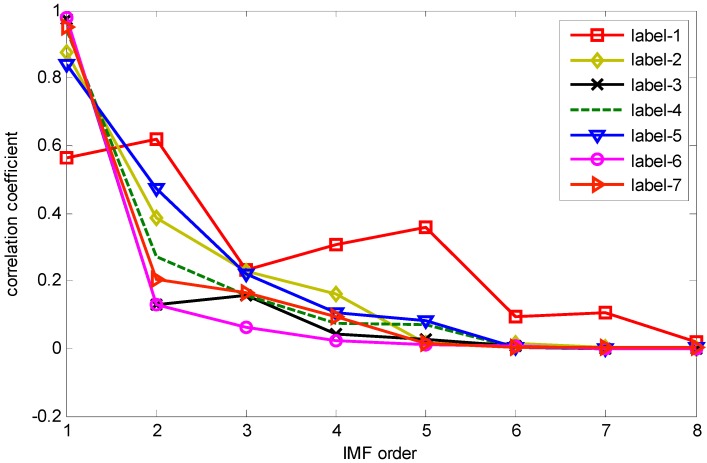
Correlation coefficient between IMF component and original vibration signal.

**Figure 3:**
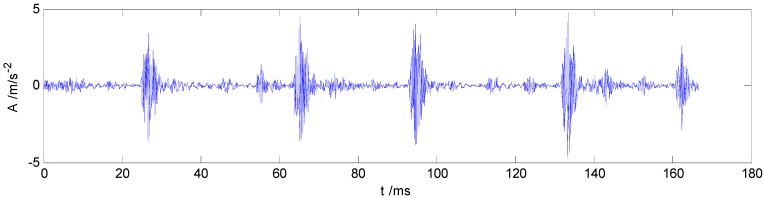
The original ORF vibration signal.

**Figure 4:**
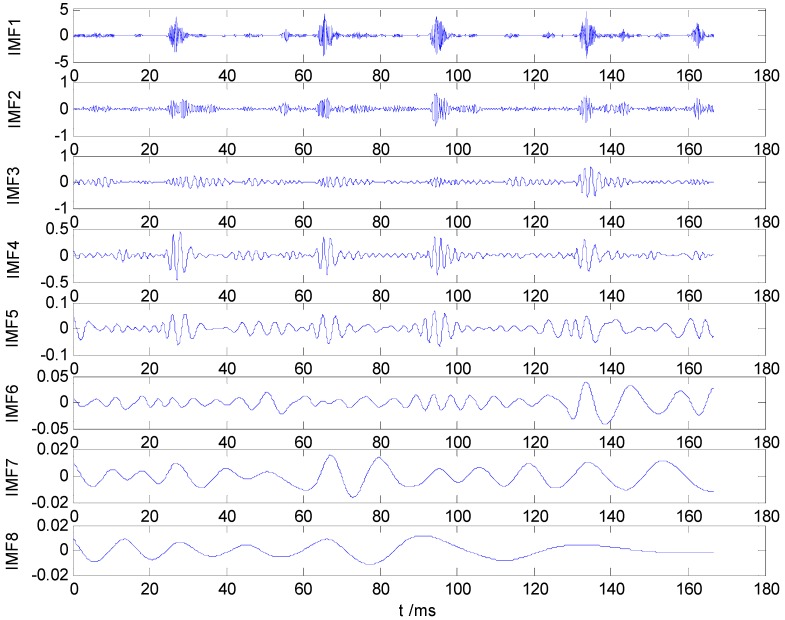
IMF1–IMF8 of ORF vibration signal.

The correlation coefficients between IMF and the original vibration signal are calculated by Equation (10). The IMFs correlation coefficient values of one sample from each label in dataset B are shown in Figure 2, which values indicate that the first five IMF components are most relevant to the original vibration signal. Therefore, IMF1–IMF5 are selected to calculate the HMS. Taking as example the REBs fault vibration signal of label 7 (ORF) in dataset B, one vibration signal sample and the first 8 IMFs from the sample are presented in Figure 3 and Figure 4, respectively.

**Figure 5:**
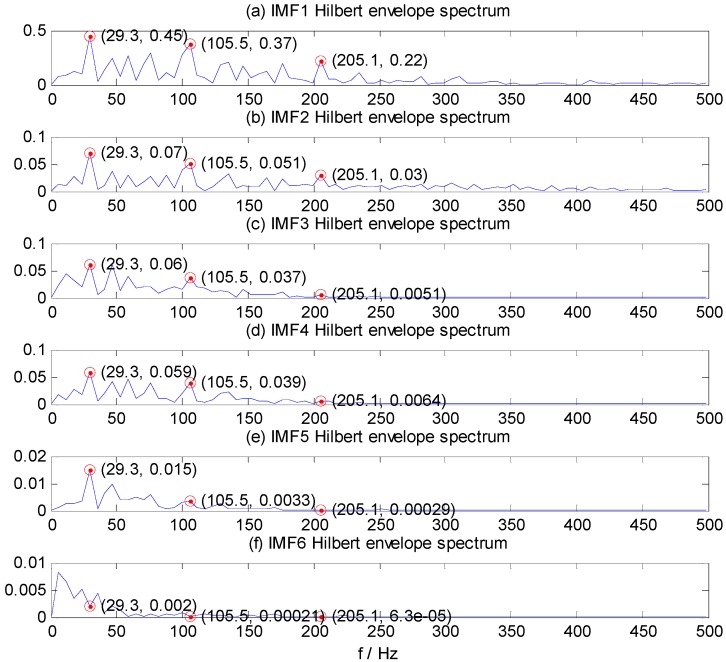
IMF1–IMF6 Hilbert envelope spectrum of ORF vibration signal. (**a**) IMF1; (**b**) IMF2; (**c**) IMF3; (**d**) IMF4; (**e**) IMF5;(**f**) IMF6.

**Figure 6:**
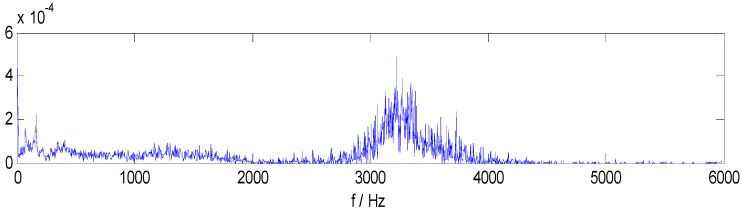
HMS of ORF vibration signal.

The bearing running frequency is 28 Hz at the machine running speed 1730 r/min, while the bearing ORF characteristic frequency is 103.4 Hz, which can be calculated from the SKF-6205-2RS bearing parameters and the roller bearing fault characteristic frequency theoretical calculation formula. Figure 5a–f shows the Hilbert envelope spectrum of IMF1–IMF6. In Figure 5a–e, we can see from the IMF1–IMF5 Hilbert envelope spectrum that there are explicit spectral lines near running frequency (29.3 Hz) and double the running frequency. Similarly, there are explicit spectral lines near theoretic fault characteristic frequency (105.5 Hz) and double the fault frequency (205.1 Hz). The HMS expresses cumulative frequency amplitude across the entire measured time period, which contains frequency characteristics of each IMF component. Under different fault conditions, therefore, HMS presents different frequency amplitude distribution characteristics. Here, we select the HMS as the preliminary feature extraction method for REBs fault types detection. The HMS of outer race fault vibration signal sample is shown in Figure 6.

The research goal here is to demonstrate the effectiveness of the proposed multi REBs faults detection model, and to illustrate the performance of the proposed method in dealing with noise. Hence, the proposed method is compared with ST-SVM and HHT-SVM models, while the ST-SVM model is based on statistical characteristics and HHT-SVM model uses HMS directly as the SVM classifier input. In the ST-SVM model, vibration signal is also decomposed into IMFs by EMD. In accordance with related research [6,17,18,24,30], five time domain characteristics and five spectrum statistical characteristics, shown in Table 3 for 1st–5th IMF components, are selected as the fault features, which means altogether 50 statistical characteristics of each sample will be calculated as the SVM classifier input vector.

**Table 3:** Statistical characteristic parameters of time domain and frequency domain.

Time–Domain Feature Parameters		Frequency–Domain Feature Parameters	
Feature	Equation	Feature	Equation
Mean value	$T_{1}=\left(1/n\right)\sum_{i=1}^{n}x\left(i\right)$	Mean value	$F_{1}=\left(1/l\right)\sum_{k=1}^{l}sp\left(k\right)$
Standard deviation	$T_{2}=\sqrt{\left(1/(n-1)\right)\sum_{i=1}^{n}{\left(x\left(i\right)-T_{1}\right)}^{2}}$	Standard deviation	$F_{2}=\sqrt{\left(1/(l-1)\right)\sum_{k=1}^{l}{\left(sp\left(k\right)-F_{1}\right)}^{2}}$
Skewness	$T_{3}=\sum_{i=1}^{n}{\left(x\left(i\right)-T_{1}\right)}^{3}/\left((n-1)T_{2}^{3}\right)$	Skewness	$F_{3}=\sum_{k=1}^{l}{\left(sp\left(k\right)-F_{1}\right)}^{3}/\left((l-1)F_{2}^{3}\right)$
Kurtosis	$T_{4}=\sum_{i=1}^{n}{\left(x\left(i\right)-T_{1}\right)}^{3}/\left((n-1)T_{2}^{3}\right)$	Crest factor	$F_{4}=\max\left|sp\left(k\right)\right|/\sqrt{\left(1/l\right)\sum_{k=1}^{l}x{\left(k\right)}^{2}}$
Crest factor	$T_{5}=\max\left|x\left(i\right)\right|/\sqrt{\left(1/n\right)\sum_{i=1}^{n}x{\left(i\right)}^{2}}$	Shannon entropy	*F_5_ =* Equation (16)
Here *x(i)* is time series of an IMF for *i* = 1,2,…,*n*, *n* is the number of data points.		Here *sp(k)* is the envelope spectrum of an IMF for *k* = 1,2,…,*l*, *l* is the number of spectrum components.	

The energy of *l* IMF spectrum components *sp(k)*, can be described as

(15) $$Energy={\sum_{k=1}^{l}\left|sp\left(k\right)\right|}^{2}$$

Shannon entropy, which measures the uncertainty of *sp(k)* is defined by

(16) $$Entropy_{sh}=-\sum_{k=1}^{l}P_{k}\log P_{k}$$

where *P_k_* is the distribution of the energy probability for each spectrum component, given by

$$P_{k}=\frac{{\left|sp\left(k\right)\right|}^{2}}{Energy}$$

The Shannon entropy is capable of determining the uncertainty and information of any distribution so that provides practical criteria for analyzing and measuring the similarity or dissimilarity between distributions of probability [6].

The classification results and optimal parameters of three REBs fault detection models for dataset A, B and C are shown in Table 4, Table 5 and Table 6 respectively. In Table 4, results show that all three of these models can achieve high classification accuracy for dataset A, while the accuracy of the ST-SVM model is slightly lower than that of other two models. However, for fault dataset B, which contains REBs faults both in different locations and at different levels of defect severity, there is a sharp decline in the performance of the ST-SVM model, while the HHT-SVM model and the HHT-WMSC-SVM model maintain good performance.

**Table 4:** Bearing fault detection results obtained by the ST-SVM, HHT-SVM and HHT-WMSC-SVM models for dataset A.

Label	ST-SVM Classification Accuracy (%)		HHT-SVM Classification Accuracy (%)		HHT-WMSC-SVM Classification Accuracy (%)	
	*C*= 0.7 *g* = 0.1		*C*= 0.1 *g* = 0.25		*C*= 2.1 *g* = 1.3 *m* = 50 *Pmin* = 355	
	Training	Testing	Training	Testing	Training	Testing
1	20/20	39/40	20/20	40/40	20/20	40/40
2	20/20	40/40	20/20	40/40	20/20	40/40
3	20/20	40/40	20/20	40/40	20/20	40/40
4	20/20	39/40	20/20	40/40	20/20	40/40
Average	100%	98.75%	100%	100%	100%	100%

The results in Table 6 show that the capability of ST-SVM model and HHT-SVM model trained by “severe” REBs fault data are weak to recognize the “incipient” REBs fault patterns. They can only recognize whether the bearing is healthy or not, but lose efficacy in the classification of different fault locations. Nevertheless, the HHT-WMSC-SVM model can achieve relatively high classification accuracy in this case.

Compared with statistical characteristics, HMS increase SVM classifier efficiency for the REBs fault classification. Furthermore, we can see that the fault classification performance of the proposed HHT-WMSC-SVM model has advantages over the HHT-SVM model, demonstrating that the WMSC method extracts the salient features in HMS that preserve most of the information related to REBs fault patterns.

**Table 5:** Bearing fault detection results obtained by the ST-SVM, HHT-SVM and HHT-WMSC-SVM models for dataset B.

Label	ST-SVM Classification Accuracy (%)		HHT-SVM Classification Accuracy (%)		HHT-WMSC-SVM Classification Accuracy (%)	
	*c* = 12.1 *g* = 0.084		*c* = 0.1 *g* = 12.3		*c* = 1 *g* = 7.02 *m* = 168 *Pmin* = 375	
	Training	Testing	Training	Testing	Training	Testing
1	20/20	39/40	20/20	40/40	20/20	40/40
2	20/20	40/40	20/20	40/40	20/20	40/40
3	20/20	35/40	20/20	40/40	20/20	40/40
4	20/20	37/40	20/20	40/40	20/20	40/40
5	20/20	34/40	20/20	38/40	20/20	40/40
6	20/20	40/40	19/20	34/40	19/20	39/40
7	20/20	36/40	20/20	40/40	20/20	40/40
Average	100%	93.21%	99.29%	97.14%	99.29%	99.64%

**Table 6:** Bearing fault detection results obtained by the ST-SVM, HHT-SVM and HHT-WMSC-SVM models for dataset C.

Label	ST-SVM Classification Accuracy (%)		HHT-SVM Classification Accuracy (%)		HHT-WMSC-SVM Classification Accuracy (%)	
	*c* = 12 *g* = 0.03		*c* = 1.8 *g* = 1.12		*c* = 3 *g* = 1.7 *m* = 78 *Pmin* = 407	
	Training	Testing	Training	Testing	Training	Testing
1	20/20	39/40	20/20	40/40	20/20	40/40
2	20/20	0/40	20/20	40/40	20/20	40/40
3	20/20	0/40	20/20	0/40	20/20	38/40
4	20/20	5/40	20/20	0/40	20/20	37/40
Average	100%	27.5%	100%	50%	100%	96.25%

**Figure 7:**
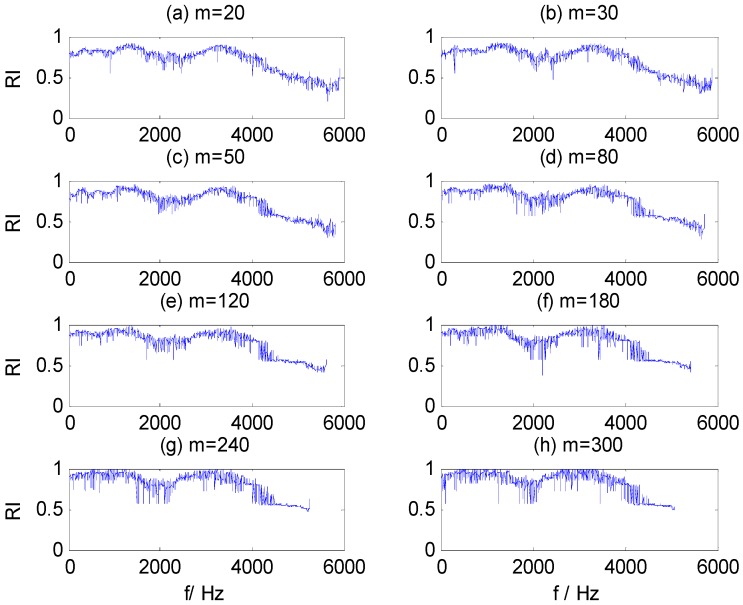
RI value sequences in different window size m. (**a**) m = 20; (**b**) m = 30; (**c**) m = 50; (**d**) m = 80; (**e**) m = 120; (**f**) m = 180; (**g**) m = 240; (**h**) m = 300.

Figure 7a–g contains the RI sequences for dataset B training samples, calculated by WMSC at different window sizes *m*, where the x-axis is the start frequency component for the sub-HMS window set and the y-axis is the corresponding RI value. The same figure illustrates that the distribution of RI values presents certain regularity at different window sizes: (1) the RI value is greater in two frequency bands, 1200–1500 Hz and 3300–3700 Hz; and (2) the waveform and change trend of the RI sequences are similar. Furthermore, the extreme RI sequence values increase and variability is aggravated with increased of window size. Appropriate values for WMSC method parameters, *m* and *Pmin*, are needed to extract optimal CFBs, from which salient HMS features are then extracted.

**Figure 8:**
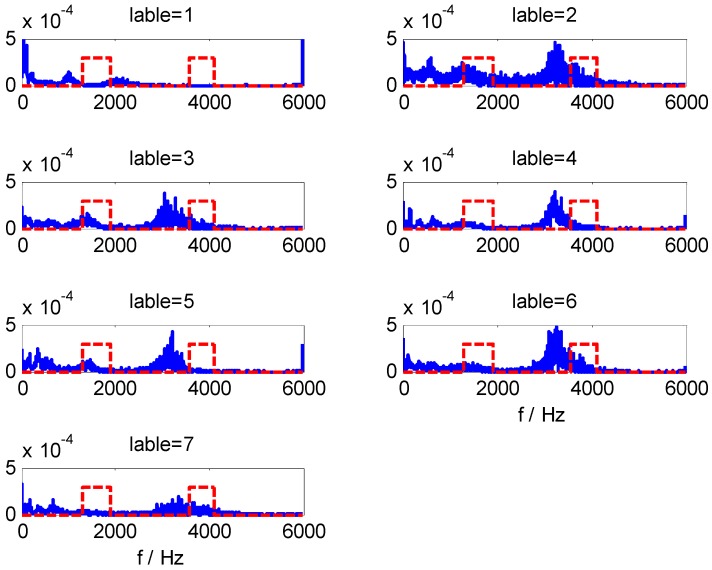
The extracted HMS-CFBs of samples in different kinds of fault types.

For data set B, we can obtain CFBs by WMSC method using the optimal model parameters *m* = 168 and *Pmin* = 375. The extracted HMS-CFBs of seven samples among different kinds of fault types are shown in Figure 8, where the HMS curve is blue and the non-zero areas along the red dotted line are the selected CFBs. Comparisons of multiple samples sets (Figure 8 shows only one sample set in different fault types) confirm the high sensitivity for fault-type identification of extracted HMS-CFBs.

**Figure 9:**
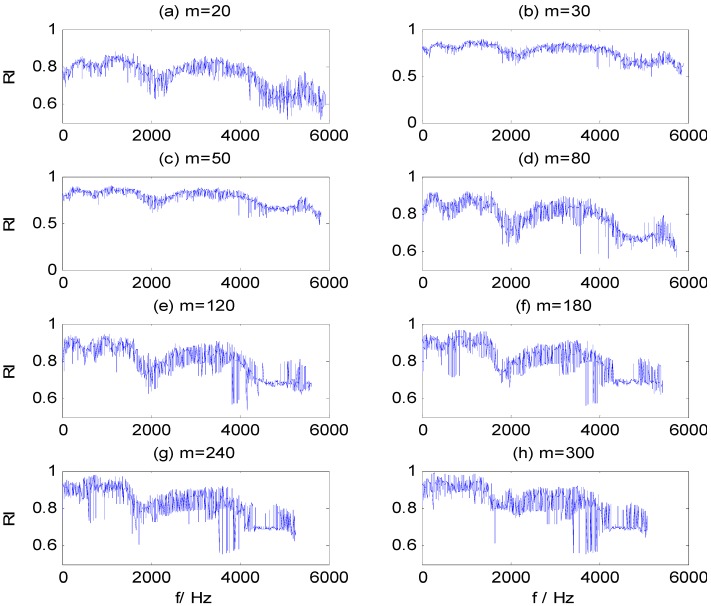
RI value sequences in different window sizes m for SNR = 5. (**a**) m = 20; (**b**) m = 30; (**c**) m = 50; (**d**) m = 80; (**e**) m = 120; (**f**) m = 180; (**g**) m = 240; (**h**) m = 300.

**Figure 10:**
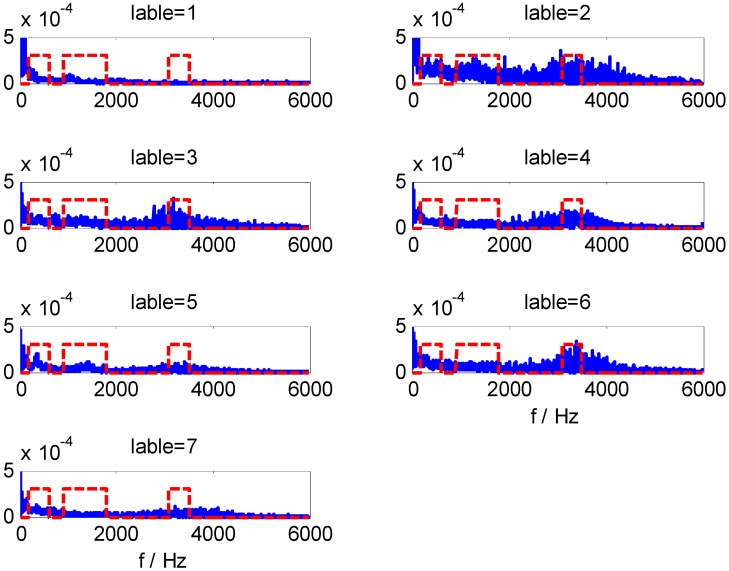
The extracted HMS-CFBs of samples in different kinds of fault types for SNR = 5.

By adding Gauss white noise to the vibration signals of dataset B at different SNR values, we further test the three models, for which Table 7 shows classification results. It can be seen that the impact on the classification results of these models is very small when SNR exceeds 10. However, when SNR is less than 10, the classification accuracy of ST-SVM and HHT-SVM models are significantly reduced with the decrease of SNR, while the HHT-WMSC-SVM model still maintains high classification accuracy. The result shows that the HMS-CFBs extracted by the WMSC method can reduce unconsidered information that is not sensitive to fault detection and improve the anti-noise ability of the model. For the training samples of dataset B with added noise at SNR = 5, the RI sequences calculated by WMSC at different window sizes *m* are shown in Figure 9a–h. The extracted HMS-CFBs of seven samples in different kinds of fault types are shown in Figure 10.

**Table 7:** Bearing fault detection results obtained by ST-SVM, HHT-SVM and HHT-WMSC-SVM models by adding Gauss white noise in different SNRs for dataset B.

SNR	ST-SVM Classification Accuracy (%)		HHT-SVM Classification Accuracy (%)		HHT-WMSC-SVM Classification Accuracy (%)	
	Training	Testing	Training	Testing	Training	Testing
3	95.71	84.64	96.42	79.64	99.28	95.00
5	97.85	87.85	95.00	84.29	99.28	98.57
7	98.57	89.64	99.28	92.86	99.28	99.28
9	98.57	88.57	100	95.35	100	98.57
11	98.57	90.35	100	96.42	99.28	99.28
13	99.20	90.00	99.28	95.71	99.28	99.28
15	99.29	92.50	99.28	97.50	100	99.64

### 4.3. Parameters Analysis of the Proposed Model

**Figure 11:**
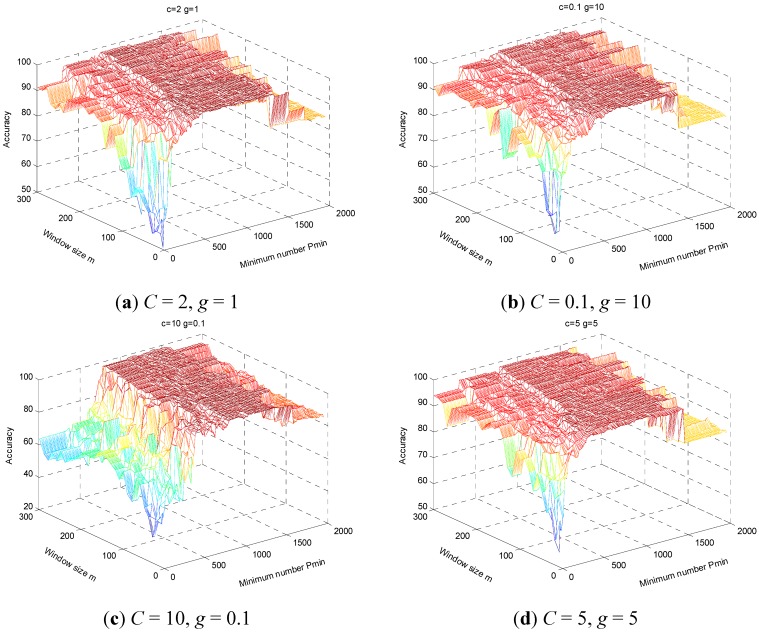
The performance of HHT-WMSC-SVM model varies with parameters (*m*, *Pmin*) under fixed parameters (*C*, *g*).(**a**) *C* = 2, *g* = 1;(**b**) *C* = 0.1, *g* = 10;(**c**) *C* = 10, *g* = 0.1;(**d**) *C* = 5, *g* = 5.

In order to analyze the effects of the parameters *m* and *Pmin* in WMSC method on fault classification capability, we test the HHT-WMSC-SVM model under different *m* and *Pmin* parameters on dataset B, adding Gaussian white noise at SNR = 5, while *C* and *g* parameters are fixed. Figure 11 displays results for the above on a three dimensional surface, where the x-axis and y-axis represent the minimum frequency points *Pmin* and window size *m*, respectively, and the z-axis represents the average classification accuracy. In Figure 11, parameters *C* and *g* are fixed to (2, 1), (0.1, 10), (10, 0.1), and (5, 5) (Figure 11a–d, respectively), the range of *m* is 10 ~ 300 and the range of *Pmin* is *m*~1998. The distribution of classification accuracy presents a strong regularity as follows: (1) the classification accuracy is usually low when *Pmin* is less than 300, rising with acceleration until the *Pmin* is about 500, and reaching maximum value between 300–800; (2) the model maintains high accuracy when *Pmin* is between 500–1300, while classification accuracy begins to decrease when *Pmin* is greater than 1500, falling to the same level as that of the HHT-SVM model. Furthermore, classification accuracy is lowest while *m* and *Pmin* are small (*m* < 150, *Pmin* < 300), because the extracted features are insufficient to effectively identify fault types, which result shows that the sensitivities to fault types of HMS vary under different frequency bands and some components may influence the effectiveness of the classifier. Improved classification accuracy demands feature extraction, which is more sensitive to the classification target, for which the WMSC method proposed here is suitable.

### 4.4. Comparison with Some Previous Works

Table 8 summarizes previous works on automated identification of REBs faults. In [23,24,32], REBs fault locations identification methods were studied, while in other literature, REBs fault locations and fault severities were diagnosed in combination. In [24], the used dataset was generated by the world pre-eminent NSF Industry/University Cooperative Research Center for Intelligent Maintenance Systems (IMS) and in [32], datasets were generated using an REBs test bench and locomotive roller bearing test bench, while in other literature, datasets were generated by the Bearing Data Center of CWRU.

**Table 8:** Comparison between this paper with some previous research in the literature for REBs fault type and fault severity classification.

Reference	Features Extraction	Classifier	Fault Types	Training/Testing Samples	Accuracy
[7]	Nine statistical parameters extracted from the paving of wavelet packets at different decomposition depths and sensitive feature selection with DET	C1: SVR C2: “one-against-one” SVM	D1: 4, HB, IRF, ORF, BF (0.007 in)z D2: 4, HB, IRF, ORF, BF (0.014 in)	120/240	C1+D1:100; C1+D2:99.58% C2+D1:97.9%; C2+D2:93.33%
[19]	LCD + fuzzy entropy	ANFIS	7, HB, IRF (0.007, 0.021 in), ORF (0.007, 0.021 in), BF (0.007, 0.021 in)	70/70	100%
[23]	Features derived from HOSA of vibration signals + PCA	“one-against all” SVM	4, HB, IRF, ORF, RF	234/150	96.98
[24]	Time–frequency domain features derived from EMD energy entropy of the first eight IMFs and statistical measurements	ANN	7, HB, IRD, ORD, RD, IRF, ORF, BF	-	93%
[25]	Time domain features(Range, absolute average, root mean square (RMS) and standard deviation)	Improved ant colony optimization (IACO)-SVM	D1: 4, HB, IRF, ORF, BF (0.007 in) D2: 4, HB, IRF, ORF, BF (0.021 in)	480/320	D1: 97.5; D2: 98.25;
[30]	Two time–domain features and two frequency–spectrum features	C1: GS-SVM C2: DE-SVM C3: ICDF-BBDE-SVM	6, IRF, ORF, BF (0.007, 0.021 in)	420/180	C1: 98.22; C2:98.28; C3:98.70
[32]	Statistical characteristics in time- and frequency–domains and Statistical characteristics of IMFs, and optimal features selected by bigger distance evaluation criteria	C1: SVM with RBF kernel C2: Wavelet-SVM with Mexican hat kernel C3: Wavelet-SVM with Morlet kernel	D1: 4, HB, IRF, ORF, BF (REBs) D2: 4, HB, IRF, ORF, BF (locomotive roller bearings)	120/80	C1+D1:91.25; C2+D1:96.25 C3+D1:97.5 C1+D2:90; C2+D2:97.5; C3+D2:98.75
[33]	Permutation entropy of IMFs decomposed by EEMD	SVM with parameter optimized by ICD	12, HB, IRF (0.007, 0.014, 0.021, 0.028 in), BF (0.007, 0.014, 0.021, 0.028 in), ORF (0.007, 0.014, 0.021)	660/990	97.91
[37]	F1-F5: SAEMD, KPCA, KFDA, KMFA, SSKMFA	C1: KNN; C2: SVM	10, HB, IRF (0.007, 0.014, 0.021 in), ORF (0.007, 0.014, 0.021), BF (0.007, 0.014, 0.021, in)	200/400	F1+C1:65.5; F2+C1:67.5; F3+C1: 70.75; F4+C1: 75.75; F5+C1: 88.0; F1+C1:88.0; F2+C2: 70.5; F3+C3: 68.25; F4+C4: 98.5; F5+C5: 100;

Note: inner race degeneration (IRD), outer race degeneration (ORD), ball degeneration (BD), distance evaluation technique (DET), Improved ant colony optimization (IACO), higher order statistics analysis (HOSA), inter-cluster distance (ICD), Local characteristic-scale decomposition (LCD), Adaptive neuro-fuzzy inference systems (ANFIS), statistical analysis and empirical mode decomposition (SAEMD), kernel principal component analysis (KPCA), kernel Fisher discriminant, analysis (KFDA), kernel Marginal Fisher analysis KMFA, semi-supervised kernel Marginal Fisher analysis (SSKMFA), K Nearest Neighbor (KNN).

Most references in Table 8 have been tested using the same bearing data set described in this article. In the references, fault features are extracted by statistical characteristics in time domain, frequency domain or time–frequency domain, following, machine learning methods are used for the purpose of fault detection. While, in this article, the entire HMS is choose as the preliminary features for REBs fault diagnosis, which is prove as a feasible solution by the experiment. Meanwhile, in order to remove the redundant and irrelevant information, a CFBs selected method WMSC method is proposed to extract the salient features from the entire HMS. Table 8 indicates that the usage of CFBs-HMS features along as feature vector yields satisfying result with the most researches. In addition, we test the anti-noise capability of WMSC method by adding Gauss white noise signal at different SNR values to the original vibration signal, which results are very positive.

## 5. Conclusions and Future Work

In this paper, a sensitive, fault-type CFBs selection method WMSC is proposed for extraction of salient HMS features. These frequency bands and their HMS features are combined with SVM to construct a hybrid REBs fault diagnosis model HHT-WMSC-SVM. The REBs defect datasets from the Bearing Data Center of CWRU were employed to verify the REBs faults classification performance of the HHT-WMSC-SVM model. Comparing experimental results with ST-SVM and HMS models, one may deduce the following:

(1) Compared to statistical characteristics, the HMS can make the fault classifier achieve higher fault recognition accuracy, especially in regard to different locations and at different levels of severity. The proposed WMSC method can extract fault type sensitive features, while filtering out redundant HMS information by the selected CFBs. In this regard, the classification performance of HHT-WMSC-SVM model surpasses that of HHT-SVM.
(2) Since CFBs are constructed according to the RI of the frequency band sub-HMS windows sets clustering results, HMS components containing noise with certain effects on classification accuracy will be discard. Adding Gauss white noise to the vibration signals, when the SNR is smaller than 10, the classification accuracy of ST-SVM and HHT-SVM models decline sharply, while the HHT-WMSC-SVM model can maintain good performance.
(3) The classification accuracy of the model is high when *Pmin* is 500–1300, which can exceed 95% under a *Pmin* range of 500–800 and an *m* range of 50–300. The preceding indicates good performance by the HHT-WMSC-SVM model across a stable range of WMSC parameters.

The combined results show that the proposed WMSC method provides a competitive alternative for preprocessing and feature extraction of REBs defect signal analysis. The HHT-WMSC-SVM model has potential for application in development of online, early REBs fault diagnosis systems. Further research may address refinement for removal of redundant components in the HMS-CFBs and other improvements. Such efforts to improve this method should incorporate effective and efficient algorithms for dimension reduction. By accurate and efficient signal diagnosis, much time and expense can be readily conserved for a range of industries.
